# Electroencephalographic Variation during End Maintenance and Emergence from Surgical Anesthesia

**DOI:** 10.1371/journal.pone.0106291

**Published:** 2014-09-29

**Authors:** Divya Chander, Paul S. García, Jono N. MacColl, Sam Illing, Jamie W. Sleigh

**Affiliations:** 1 Department of Anesthesiology, Perioperative and Pain Medicine, Stanford University School of Medicine, Stanford, California, United States of America; 2 Department of Anesthesiology, Atlanta VA Medical Center/Emory University, Atlanta, Georgia, United States of America; 3 Department of Anaesthesia, Waikato Clinical School, University of Auckland, Hamilton, New Zealand; McLean Hospital/Harvard Medical School, United States of America

## Abstract

The re-establishment of conscious awareness after discontinuing general anesthesia has often been assumed to be the inverse of loss of consciousness. This is despite the obvious asymmetry in the initiation and termination of natural sleep. In order to characterize the restoration of consciousness after surgery, we recorded frontal electroencephalograph (EEG) from 100 patients in the operating room during maintenance and emergence from general anesthesia. We have defined, for the first time, 4 steady-state patterns of anesthetic maintenance based on the relative EEG power in the slow-wave (<14 Hz) frequency bands that dominate sleep and anesthesia. Unlike single-drug experiments performed in healthy volunteers, we found that surgical patients exhibited greater electroencephalographic heterogeneity while re-establishing conscious awareness after drug discontinuation. Moreover, these emergence patterns could be broadly grouped according to the duration and rapidity of transitions amongst these slow-wave dominated brain states that precede awakening. Most patients progressed gradually from a pattern characterized by strong peaks of delta (0.5–4 Hz) and alpha/spindle (8–14 Hz) power (‘Slow-Wave Anesthesia’) to a state marked by low delta-spindle power (‘Non Slow-Wave Anesthesia’) before awakening. However, 31% of patients transitioned abruptly from Slow-Wave Anesthesia to waking; they were also more likely to express pain in the post-operative period. Our results, based on sleep-staging classification, provide the first systematized nomenclature for tracking brain states under general anesthesia from maintenance to emergence, and suggest that these transitions may correlate with post-operative outcomes such as pain.

## Introduction

Both sleep and anesthesia have been tracked in the human electroencephalogram (EEG) since the 1950s [1]–[3], but a staging nomenclature based on specific EEG features has only been well-developed for natural sleep [4], [5]. While there are also stereotyped EEG features, such as a shift in the spectrum toward lower frequencies (<14 Hz), that correlate with anesthesia-induced unresponsiveneses (the historical clinical marker of anesthetic adequacy) [6]–[11], a standardized nomenclature for anesthetic maintenance and emergence has never been universally accepted by clinicians. Recent focus has been placed on the spatial and temporal distribution of these frequency changes [12]–[15], and identifies an anteriorization of lower frequency power that correlates with the behavioral transition to loss of consciousness [8], [9], [16], [17]. Studies in which volunteers have been exposed to slowly changing doses of propofol have described spectral power changes upon emergence that appear to be the inverse of induction, i.e. a decrease in power in these low frequency oscillations over the frontal EEG, followed by an increase in higher frequencies as the patient becomes behaviorally responsive to mild stimuli [8], [9]. This is in contrast to the termination of natural sleep, which is typically preceded by cyclic transitions into progressively longer episodes of cortical activation [18]. Waking usually occurs from rapid eye movement (REM) sleep (reviewed in Steriade [19]).

Although sleep and anesthesia are not the same, significant overlap in neurotransmitters, circuitry and electrical patterns do exist [20], [21], suggesting that anesthetics may achieve part of their hypnotic effect by acting on the normal sleep and arousal systems of the brain [22]. Pharmacologic enhancement of inhibitory signaling, mediated via the gamma aminobutyric acid type A (GABA_A_) receptor, underlies the mechanisms of most commonly used anesthetic agents [23], [24]. Similarly, a physiological GABAergic state also contributes to the thalamocortical oscillations characteristic of non-REM sleep [25], while pharmacologic inhibition of the GABA_A_ receptor reverses sleepiness in hypersomnic patients [26]. It has been shown that sleep deprivation potentiates the efficacy of anesthetics [27], and these effects can be partially reversed by antagonists to adenosine, an endogenous ligand that accumulates during sleep pressure [28]. Furthermore, activation of subcortical endogenous arousal pathways (e.g. thalamus, basal forebrain, hypothalamus, brain stem) can reverse anesthesia in some animal models [29]–[36], significantly blurring the distinction between sleep and anesthesia. Perhaps most importantly, both non-REM (NREM) sleep and general anesthesia exhibit similar synchronization and slowing of the EEG [37], [38]. Not surprisingly, the same range of lower frequency oscillations that indicate loss of consciousness are also used to separate natural sleep into specific stages [4], [5], [39], [40]. Thalamocortical oscillations in the alpha band (8–14 Hz) are the electroencephalographic hallmark of the loss of perceptual awareness in synchronized sleep [41]. Generated by the reticular nucleus of the thalamus [42], these oscillations consist of waxing and waning of electrical potentials, and are often referred to as sleep spindles [43]. As sleep deepens, spindles are progressively reduced and replaced by slower oscillations (0.5–4 Hz) [44], [45]. In contrast, REM sleep is characterized by profound inhibition of motor output, abolition of these low-frequency oscillations, and an EEG resembling the aroused brain [46]. Although the different sleep stages were arbitrarily and heuristically defined, based on EEG patterns described above [5], [47], they have been successfully used by clinicians and researchers to relate sleep physiology to functional neuroanatomy [48], consolidation of memory [49]–[51], and sleep disorders [52], [53].

We therefore designed a study with three objectives. The first was to catalogue and define a standardized nomenclature for the EEG during anesthestic maintenance, analogous to that used to describe natural sleep. The second was to characterize the evolution of the human EEG during emergence from general anesthesia during surgery, to determine if there was a single common path, or multiple pathways to re-establishing conscious awareness and interaction with the outside world. Our final goal was to determine if the path by which the patient emerged had any relationship to the quality of a patient's recovery.

We recorded the frontal EEG from 100 human subjects in the operating room, undergoing routine orthopedic surgery with general volatile anesthesia, prior to loss of consciousness, through the maintenance and recovery from anesthesia, until patients were able to be taken safely to the recovery room. Using spectral processing techniques, we propose a nomenclature of four basic EEG patterns, based on the relative dominance of low frequency oscillatory patterns seen during maintenance of general anesthesia (i.e. a degree of hypnosis considered to be commensurate with unconsciousness). We also identified four stereotypical trajectories that describe the progression toward conscious awareness during emergence. These emergence patterns do not consistently follow the averaged patterns of volunteers undergoing slow infusions of propofol described in prior studies [8], [9], but in some instances do share similarities with sleep stages that precede awakening. Further, we noted that the trajectory taken to re-establish conscious awareness is not correlated to the brain state at the cessation of the anesthetic (End Maintenance), but the path taken to re-establishing awareness does appear to have some correlation with a subject's subsequent level of sedation and post-operative pain. Our work suggests that the central nervous system may not always re-establish connectivity in a canonical sequence after unconsciousness produced by general anesthesia during surgery. We hypothesize that these less typical emergence sequences under anesthesia may predispose patients to undesirable wake-ups, in a similar way that parasomnias are exacerbated by disrupted sleep architecture [54].

## Results

### Patterns during maintenance in the human frontal electroencephalograph can be divided into four (4) basic spectral variants

**Figure 1:**
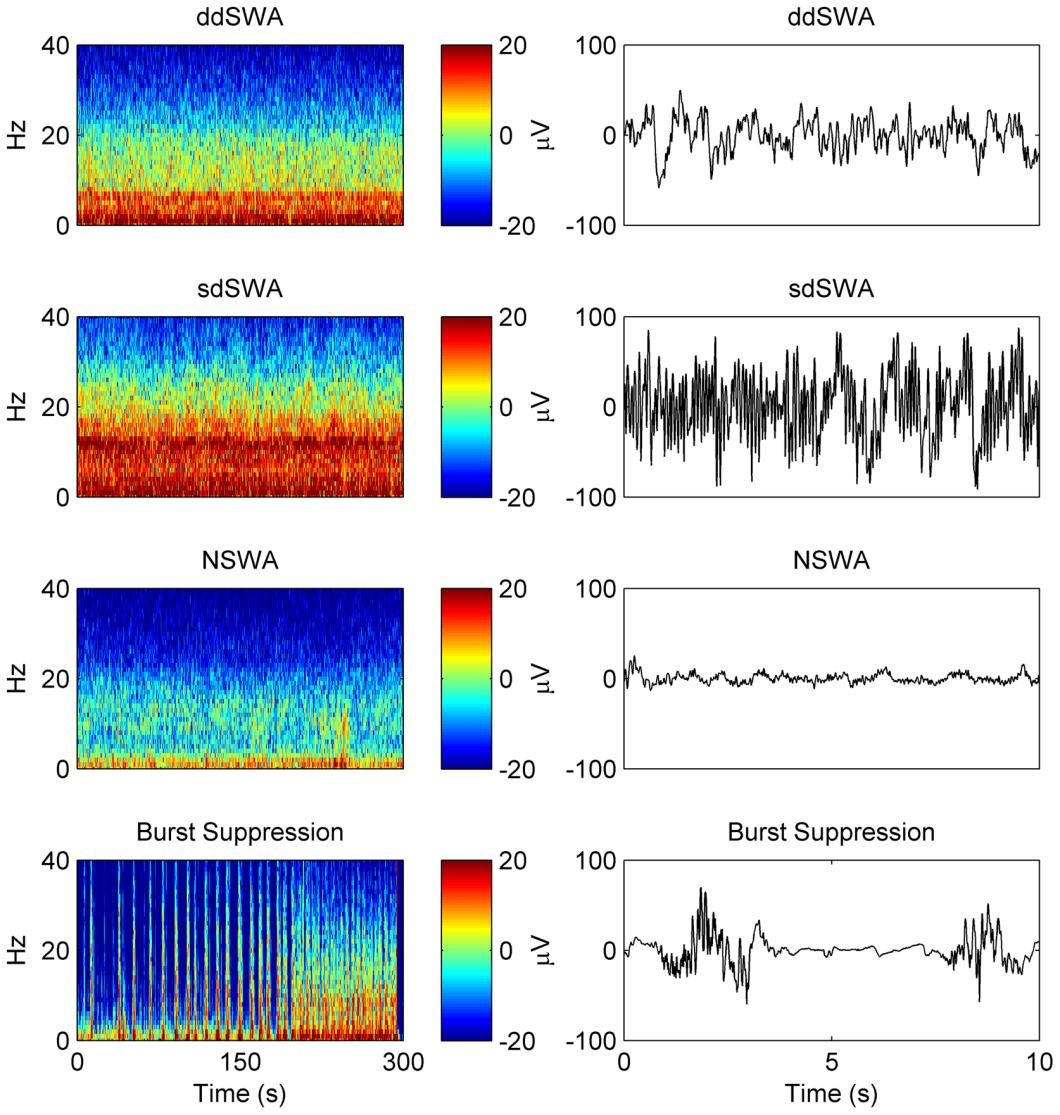
Spectral patterns at the end of maintenance under sevoflurane general anesthesia comprise 4 basic patterns. ‘Slow-Wave Anesthesia’ (SWA) is a spectral pattern in which there is both high delta and spindle power (>7 dB). Panel (A) shows the more common variant, or subclass, of the SWA spectrogram, calculated over 5 minutes prior to turning off the anesthetic, in which delta power is higher than spindle power (point #1 below the diagonal in Figure 2B) termed delta dominant SWA, or ddSWA. An example 10 second raw EEG tracing is take from this period and shown in the right column. Panel (B) is an SWA spectral variant/subclass in which spindle power is higher than delta power, termed spindle-dominant SWA, or sdSWA (point #2 above the diagonal in Figure 2B). Finally, a small subset of patients (5%) showed low amplitude power in both the spindle and delta frequency bands (C). We termed this pattern non Slow-Wave Anesthesia, or NSWA (point #3 in Figure 2B). For completeness, the spectrogram in panel (D) reflects a deeper anesthetic maintenance pattern, burst suppression, transitioning into a ddSWA pattern at approximately 1.5 minutes prior to End Maintenance. The representative 10 second EEG to its right is taken from the burst suppression period.

**Figure 2:**
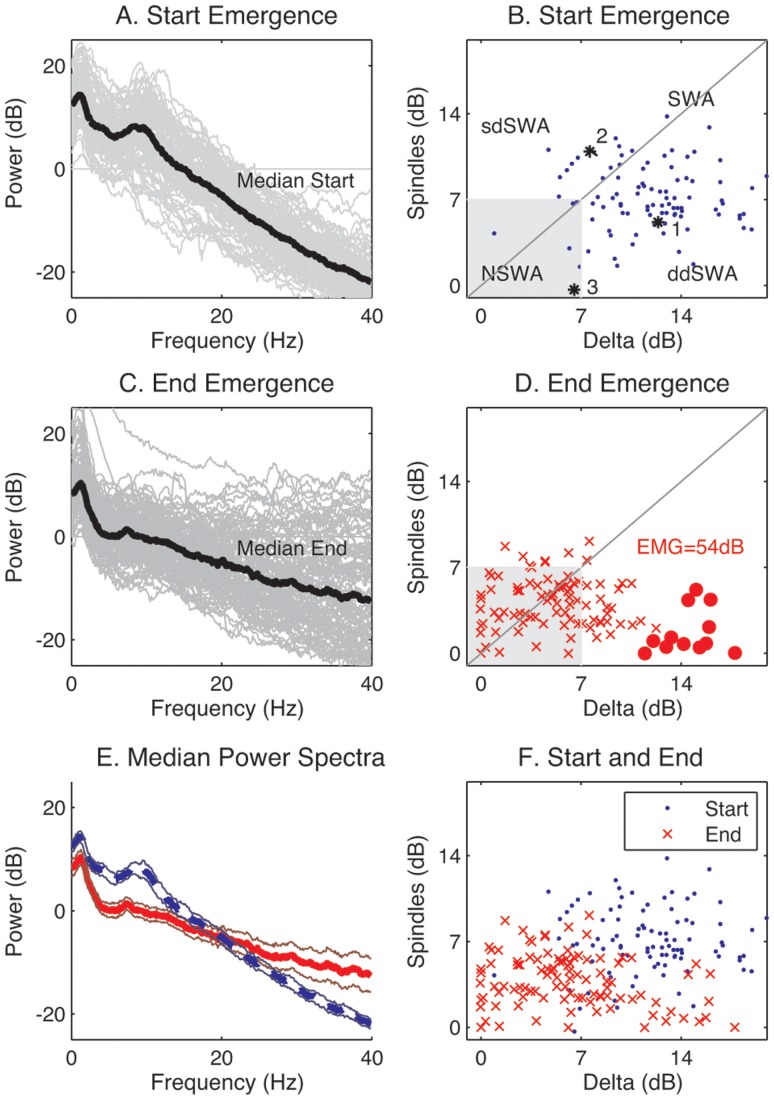
Emergence from general anesthesia is characterized by a loss in power in the slower spindle and delta frequency bands, and a recovery of power in the higher frequency bands. Power spectra and scatterplots of delta and spindle power (dB) for all patients, at the start (2A, 2B) and the end (2C, 2D) of emergence. The thick black lines are the median of the power spectra at each frequency for all patients. The median power spectra and 95% confidence intervals of the median, at the start and end, are shown in 2E. Patients were separated into two main spectral classes by thresholding spindle and delta power at 7 dB, whose boundary is marked by the gray box (see also Figure S1). Thus, the most common pattern, seen in 95% patients at Start Emergence, is characterized by high power (>7 dB) in both frequency bands, which we define as ‘**Slow-Wave Anesthesia (SWA)**’; the alternate pattern, defined as ‘**Non Slow-Wave Anesthesia (NSWA)**’, represents the 5% of patients that fall within the gray box. The fuzzy clusters in 2B are defined in the text: SWA  =  Slow-Wave Anesthesia, ddSWA  =  delta-dominant Slow-Wave Anesthesia, sdSWA  =  spindle-dominant Slow-Wave Anesthesia, NSWA  =  Non Slow-Wave Anesthesia (gray box). Red circles in 2D are those patients with a high delta power at End Emergence. All except one subject with high delta at End Emergence also had an EMG>40 dB. 2F is the same as 2B and 2D superimposed for ease of visual comparison.

In order to define a nomenclature for anesthetic maintenance, we analyzed the relative contribution amongst major oscillatory features found in the power spectra of the frontal electroencephalogram (EEG) of 100 patients undergoing routine orthopedic surgery under general anesthesia. The time at which the anesthetic was turned off was defined as the ‘End Maintenance’ or ‘Start Emergence’ point. A spectrogram of EEG data from a single frontal EEG electrode for 5 minutes prior to End Maintenance was computed for each individual patient. Visual inspection revealed clear peaks in the spectrogram within the delta and alpha bands of some patients, but these peaks showed considerable variation across the population (Figure 1A-D). The power spectra and a scatterplot of delta power against the oscillatory component of alpha power, computed over an 8 second EEG window (see Methods) for all 100 patients at Start Emergence, are shown in Figures 2A and 2B. The oscillatory component of alpha power was quantified by measuring the height of the alpha peak above the underlying broadband activity [55]. This has the advantage that it specifically targets the narrowband oscillatory component of this peak, and is less influenced by the underlying broadband (1/*f*) components of the EEG, making the alpha oscillatory power more orthogonal to the delta power. In this study we have chosen to borrow the term ‘spindles’ from the sleep literature to describe the specific oscillatory component in the alpha range from the overall composite alpha power [5]. Although the scatterplot in Figure 2B does not readily segregate into discrete clusters, we followed statistical convention and separated the patients into two main spectral classes by thresholding spindle and delta power at 7 dB. Because the bi-variate data appears to follow a unimodal distribution, the 7dB theshold reflects the boundary below which only 5% of the Start Emergence data lay, and the region within which most of the End Emergence data lay (Figure S1). Thus, the most common pattern, seen in 95% patients at Start Emergence, is characterized by high power (>7 dB) in both frequency bands, which we define as ‘**Slow-Wave Anesthesia (SWA)**;’ this includes patients above the 7 dB boundary marked by the gray box. Some patients have a relatively equal distribution of spindle and delta power during SWA, lying on or near the unity diagonal (Figure 2B). However, it was more common in this patient sample for delta power to be greater than spindle power during SWA. This is represented by the preponderance of points lying below the diagonal in Figure 2B. We therefore propose the term ‘**delta-dominant Slow-Wave Anesthesia**’ (**ddSWA**, Figure 1A) to describe this sub-classification. Conversely when spindle power is greater than delta power (i.e. points above the diagonal, Figure 2B), we propose the term ‘**spindle-dominant Slow-Wave Anesthesia**’ (**sdSWA**, Figure 1B). In this sample, we noted a continuum of relative spindle and delta power between patients, and even within patients during the course of their anesthetic. Because of this we have purposely left the terms sdSWA and ddSWA somewhat loosely defined – they are convenient labels that indicate a patient (in SWA) has relatively stronger spindle or delta waves. These plots are generated over a short snap-shot in time; thus, a patient in a ddSWA pattern may gradually lose delta power and transition into an sdSWA pattern over the course of an anesthetic. Five percent (5%) of patients had Start Emergence EEGs with a very low spindle and delta power (<7 dB) – we termed these indeterminate patterns “**Non Slow-Wave Anesthesia**” (**NSWA**, Figure 1C). An example of another common maintenance pattern, that of burst suppression (transitioning into SWA) is shown in Figure 1D, for as complete a description as possible of spectral patterns encountered during surgical anesthesia. This pattern is more typically seen post-induction of general anesthesia (i.e. just after loss of consciousness is achieved with a bolus dose of propofol), but may be seen during the entire course of the maintenance phase of anesthesia.

### Emergence from general anesthesia is characterized by a shift from a slow-wave pattern (SWA) to a more uniform distribution of power across all frequency bands

In order to characterize salient features of the EEG at the start (i.e. turning off anesthetic) and end of emergence (i.e. responsiveness to standard verbal stimulation), the power-spectral density (PSD) plots for individual patients were compared in each condition. The plots in the left column of Figure 2 show the power spectra for all patients (gray), derived from the 8 second EEG segments at the start (Figure 2A) and end of emergence (Figure 2C). There is considerable variation in individual patient spectral power, encompassing a 30 dB range (equivalent to an approximately five-fold variation in raw voltage) at both Start and End Emergence, with greater spread in the higher frequency bands (>14 Hz) during End Emergence. The thick black line in both Figures 2A and 2C are the median power spectra for each condition. Distinct peaks are present at Start Emergence/End Maintenance in the delta and spindle frequency bands. At End Emergence, the spindle peak is lost, and the delta peak diminishes in amplitude. The scatterplots in Figures 2B and 2D, in which delta and spindle power are plotted against one another for each patient, clearly show the downward shift in power in both these frequency bands (from 2B to 2D). There is a small subset of patients (12%) at End Emergence that maintains high delta power (red dots in Figure 2D); this subset is also the group that showed high EMG at End Emergence (>40 dB) suggesting that the high delta power is actually broad-band contamination of the EEG with frontalis muscle activity rather than true frontal cortical activity. For ease of comparison, the shifts in median power spectra at Start (blue) and End Emergence (red) are shown in Figure 2E, while the downward shift in spindle-delta power at Start (blue) and End Emergence (red) is summarized in Figure 2F. As a whole, the median power spectra at End Emergence demonstrates a more uniform distribution of power in the 20–40 Hz range, consistent with recent studies in human volunteers receiving propofol infusions [8], [9], but with more variability as evidenced by broader confidence intervals.

### Evolution of trajectories from Start Emergence to End Emergence vary by patient

**Figure 3:**
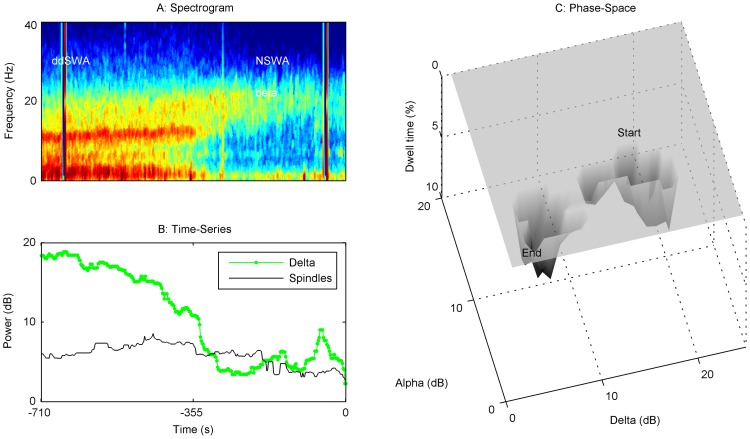
Emergence Trajectory 1, SWA → NSWA. The upper left panel (A) is the spectrogram from Start Emergence (measured in negative seconds) to End Emergence (time 0 seconds) for a representative patient (#81); this also corresponds to the time of anesthetic wash-out from the brain. Below it is a time series (B) that quantifies spindle and delta power (dB) over the same emergence period. To the right is a dwell time state-space plot (C). The evolution of spindle and delta power over the emergence trajectory from Start Emergence (upper right) to End Emergence (lower left) is shown where the depth of the contour (y-axis) reflects the time spent in each pixel of the state, as a percentage of the total emergence time. At the start of emergence, this patient remained for a signficant period in a slow-wave state (SWA) characterized by higher delta to spindle power ratio (ddSWA). There was a relatively abrupt transition period of approximately 60 seconds, to a second attractor characterized by lower delta and spindle power (NSWA) prior to waking-up. In our sample, 23% of patients had a similar trajectory.

**Figure 4:**
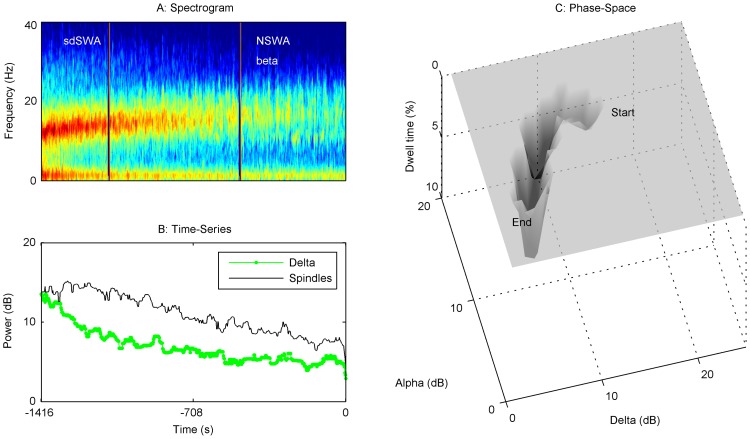
Emergence Trajectory 2, SWA→NSWA, continuous progression. The spectrogram (A) and time series (B) from Start Emergence to End Emergence for a representative patient (#36) are shown. To the right is dwell time state-space plot (C). This patient started in a slow-wave state (SWA). Over approximately 1.5 minutes, the patient transitioned into a state characterized by higher spindle power (sdSWA), and then showed a progressive decrease in delta and spindle power towards the NSWA region over about 20 minutes before waking. There was a converse increase in beta power as alpha/spindle power was diminished. No single deep attractor in state-space characterizes this patient's emergence path until reaching NSWA prior to wake-up. In our sample, 20% of patients had a similar trajectory.

**Figure 5:**
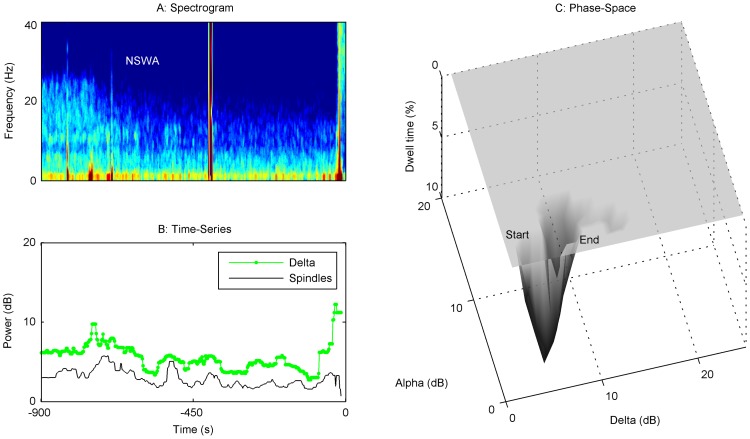
Emergence Trajectory 3, NSWA→Wakefulness. The spectrogram (A) and time series (B) from Start Emergence to End Emergence for a representative patient (#19) are shown. To the right is a dwell time state-space plot (C). This patient started and ended in a state characterized by a non slow-wave state (NSWA) attractor prior to waking up. The duration of emergence was long (15 minutes), and the attractor did not move significantly. In our sample, 16% of patients had a similar trajectory.

**Figure 6:**
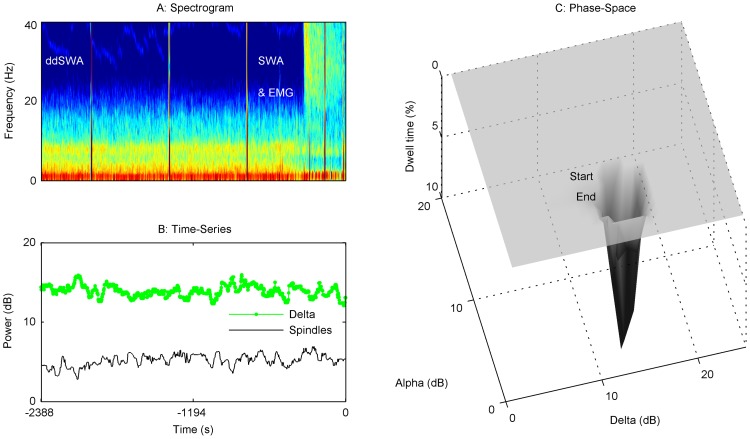
Emergence Trajectory 4, SWA→Wakefulness. The spectrogram (A) and time series (B) from Start Emergence to End Emergence for a representative patient (77) are shown. To the right is a dwell time state-space plot (C). This patient started and ended in a state characterized by a slow-wave state (SWA) attractor prior to waking up. Patients who woke up this abruptly from a slow-wave state of anesthesia were more likely to experience high pain in recovery. In our sample, 31% of patients had a similar trajectory.

Figure 2 captures a static image of the state of the brain at the start and end of emergence. While a shift from one average state to another can be seen in Figure 2E, the variability of individual patient PSDs and spindle-delta power scatterplots suggests that the path from Start to End Emergence may not be identical or stereotyped. Clinically, this is often reflected in the length of time a patient takes to emerge from general anesthesia. We chose not to average patient trajectories, and instead looked for graphical methods to describe changes for individual patients. This involved construction of an ‘emergence trajectory’ for each patient. A spectrogram from Start to End Emergence was first calculated (Figure 3–6A), and a time series data of spindle and delta power extracted from the power spectrum (Figure 3–6B). Finally, we applied a dynamical systems approach to the data, creating a corresponding 3-D ‘dwell time plot’ for each patient's emergence trajectory (Figure 3–6C). These graphically depict how the relative spindle and delta power changes for each patient in response to the progressive decline in both anesthetic drug concentrations and the residuum of surgical nociception from the time the anesthetic is turned off until the patient responded to voice. In these ‘phase-space’ plots, spindle power is reflected on the y-axis, delta power on the x-axis, and the z-axis reflects the time spent in each ‘pixel’ of state-space as a percentage of the total emergence time (“dwell time”). From these surfaces, we can see that a continuous, progressive decrease in spindle and delta power was **not** the usual pattern of emergence. Instead the majority of patients tended to follow a more “punctated equilibrium” pattern, occupying restricted regions of the state-space for extended periods of time, before relatively rapidly moving to other areas of phase space, or to the waking state. At first approximation, these areas appear to functionally act as point attractors [56]. At Start Emergence, most (95%) of these point attractors can be classified as SWA, or a sub-classification (sdSWA or ddSWA), while at End Emergence, the majority (59%) are classified as NSWA. The path from Start Emergence to End Emergence took variable trajectories. These were roughly classified by the presence of one or two attractors, whether these attractors lay in the SWA or NSWA regions, and whether the transition to a new state was rapid or gradual through the phase-space. Figure 3 demonstrates a common emergence trajectory (23% of patients). It shows an initial attractor in the SWA region, which steps abruptly to a second attractor in NSWA space, followed by arousal. The second trajectory (20%) is similar to the first except it shows a more gradual transition between SWA and NSWA state space (Figure 4). Figure 5 describes a third trajectory (16%), a single attractor in NSWA, which reflects a patient who spends nearly the entire duration of emergence in NSWA before responding to voice. The fourth trajectory (31%) is characterized by a patient who woke up directly from SWA (Figure 6). About 10% of patients fell into indeterminate categories. We would note that during emergence many patients who start emergence in the ddSWA go on to lose their delta power and gained spindle power during their emergence; this resulted in nearly half (49%) of patients spending more than 25% of their time in the sdSWA region. This transition pattern is analogous to that seen in the progression from deep to lighter natural NREM stage 2 sleep. At the other extreme, 27% patients spend no time in the sdSWA region at all, as if NREM stage 2 sleep was skipped during the process of waking.

### Evolution of trajectories from Start Emergence to End Emergence are correlated to nociceptive state and level of consciousness upon waking

**Figure 7:**
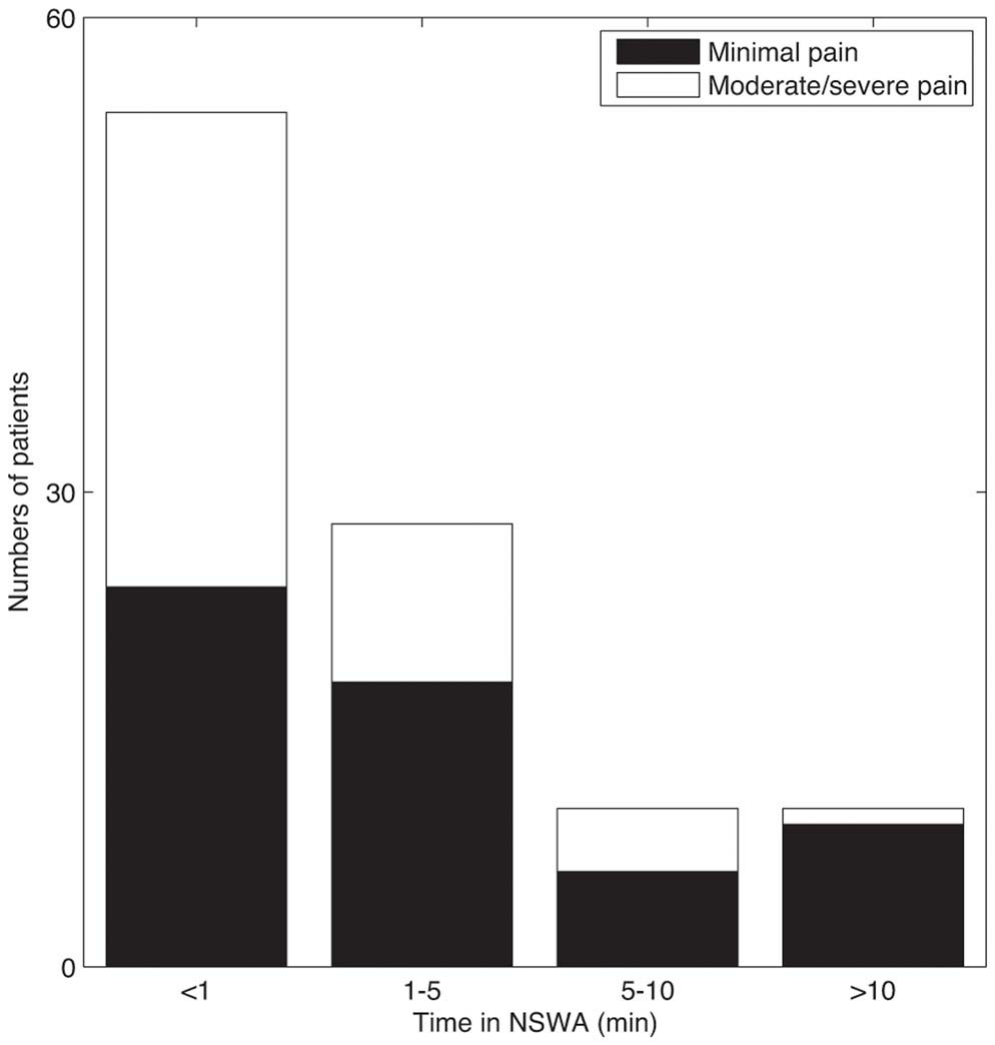
Increased time spent in a non slow-wave state (NSWA) during emergence is correlated with a lower nociceptive state. Patients had a variable duration of emergence from the time the anesthetic was turned off, ranging from 2 minutes to more than 10 minutes, as well as a variable dwell-time in the state of NSWA. When divided into 4 groups based on NSWA dwell-time, patients that spent more time in NSWA prior to emerging were more likely to have minimal pain (PACU-pain  = 0, Table 1). Of the group that spent>10 minutes in NSWA prior to wake-up, 90% had minimal pain. Of the group that spent less than a minute in NSWA prior to wake-up, 44% had minimal pain (*p* = 0.037, Chi-squared test).

**Figure 8:**
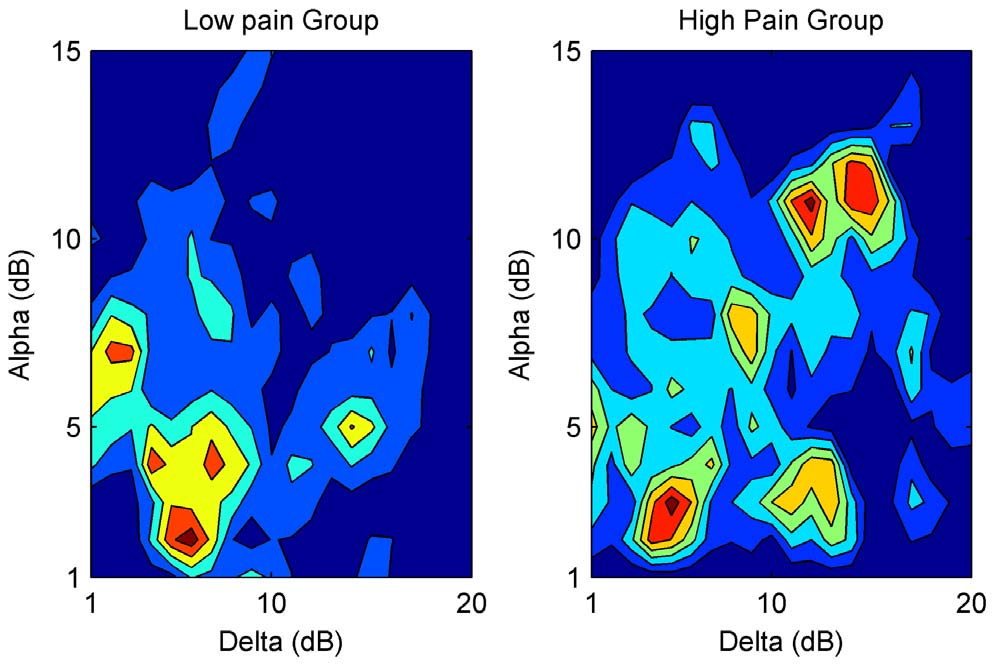
Patients with low pain scores (0) spend more time in a non slow-wave (NSWA) pattern during emergence while patients with higher pain scores (2) spend more time in a slow-wave pattern (SWA). This is reflected in the averaged trajectories through the spindle-delta state space for the low (left panel) and high pain (right panel) groups. The heat map from blue to red reflects least to most time.

We next examined whether there was an association with, the relative time spent in SWA vs. NSWA, and the *quality of emergence*. Two post-anesthesia scores were used to quantify level of consciousness and post-operative pain (Table 1). Because sedation can interfere with reported pain, we measured pain heuristically by combining self-reported pain score with non-reported signs of distress. We found an association between lower pain scores and the relative amount of time spent in NSWA during emergence (Figure 7). Those who spent a long time in a non slow-wave state prior to wake-up were more likely to waken with minimal pain (PACU-Pain  = 0) than those who woke directly from a slow-wave state (*p* = 0.037, Chi-squared test; *p* = 0.0017 for delta power distribution, *p* = 0.05 for alpha power distribution; Kolmogorov-Smirnov test). This is reflected in the averaged dwell-time contour map for the low and high pain groups (Figure 8).

**Table 1:** Level of Consciousness and Pain.

**PACU-Cons Score**	
**0**	Confused
**1**	Quickly alert (Ramsay score = 2 at 15 minutes)
**2**	Somnolent (Ramsay score = 3 or 4 at 15 minutes)
**PACU-Pain Score**	
**0**	Minimal (NRS = 0–3 and relaxed)
**1**	Moderate (NRS = 4–8 and relaxed or going back to sleep)
**2**	Severe (NRS = 8–10 or NRS>4 and signs of distress)

PACU  =  Post anesthesia care unit, Cons  =  consciousness, NRS  =  numerical rating scale

### Associations amongst patient variables with delta and spindle power

The delta power at the start of emergence was negatively correlated with age (*r* = −0.44, *p*<0.0001), and positively correlated with the C_e_MAC (*r* = 0.24, *p* = 0.015). Combined in a multiple regression model, these two variables explained 21% of the variability in delta power. Addition of anesthetic agent, regional block and gender insignificantly increased this to 24%. Delta power was not correlated with C_e_Opioid. The spindle power was also negatively correlated with age (*r* = −0.34, *p* = 0.0003) and with duration of operation (*r* = −0.29, *p* = 0.002). When combined with the C_e_MAC, the model explained 23% of the variation in spindle power. However older patients were also more likely to have longer operations (*r* = 0.25, *p* = 0.01), and receive less volatile anesthetic drug (*r* = −0.19, *p* = 0.05).

### Associations amongst patient variables, emergence time, and post-operative pain

Emergence time was positively correlated with the duration of operation (*r* = 0.24, *p* = 0.014), and weakly influenced by the C_e_MAC (*r* = 0.18, *p* = 0.07). Consistent with prior studies [57]–[60], male patients were slower to emerge (*p* = 0.02) than females. In this study, we found that different volatile drugs had no significant influence on emergence time, but the number of patients receiving desflurane or isoflurane was small. The multivariate model of emergence time is given below (the presence of a regional block is coded as 1, the absence as 0; the difference between the genders was included as a categorical variable [1/0]).
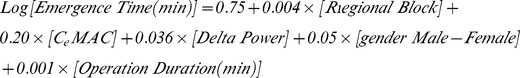
(1)


Although the model is statistically significant, these explanatory variables are not a very good explanation of the variation in emergence time, as the *r* ^2^ value was only 0.16.

When combined in the logistic regression model, the effect site concentration of opioid at the start of emergence, expressed in fentanyl equivalents (C_e_Opioid), duration of NSWA, and male gender were the most significant associations with postoperative pain. A higher opioid concentration was associated with more pain. We attribute this unusual observation to the fact that patients who were expected to have more painful operations would have been given more (but presumably not enough) opioids intraoperatively. This model had a modest predictive capability (area under ROC  = 0.71), but again the strength of association was quite weak (*r* ^2^ = 0.11).
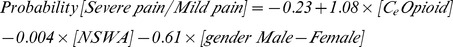
(2)


### Associations amongst patient variables and different EEG patterns of emergence

The heterogeneity in patterns of emergence seem to be largely determined by unknown intrinsic factors, and are not strongly related to the common clinical explanatory variables. We found no significant associations between the EEG emergence pattern group and simple clinical and demographic variables – namely post-operative pain, type of volatile anaesthetic drug, gender, the presence of a regional block, operation duration, or the C_e_MAC. However those patients that started in SWA, but then spent a period of time in the NSWA attractor before transitioning to wakefulness tended to be younger (mean(SEM) 43(4) years vs. 58(3) years, *p* = 0.015) and have a higher C_e_Opioid (0.80(0.09) vs. 0.44(0.08) ng/ml fentanyl equivalents, *p* = 0.04), than those who jumped directly from a SWA state to wakefulness. Thus the relationship between C_e_Opioid, postoperative pain, and EEG emergence pattern, is complex and remains to be fully elucidated in larger studies.

### EMG Activation During Emergence

The BIS EEG monitor attributes the power (dB) in the frequency range 70–110 Hz to the frontalis EMG signal, because it lies above the frequency band at which there is significant scalp EEG power. Typically the EMG power is low during general anaesthesia, and then rather abruptly increases in a stepwise fashion at various points during the emergence period. Often there are no overt clinical signs of an increase in muscle tone, or any gross motor movement. After examination of the EMG records, we chose the point at which the EMG power rose above 40 dB as a time point that reflected the activation of the EMG with reasonable face validity. The most striking feature was that the activation of the EMG often had quite a different time course to the EEG changes. The interval between EMG activation and End Emergence approximately followed an exponential probability distribution. For a quarter of the patients, their EMG became activated coincident with their waking (response to voice). Another one-half of patients woke within 74 seconds of EMG activation. However, a quarter of patients had a prolonged interval of at least 334 seconds between EMG activation and waking. The strongest correlations were with duration of operation (*r* = 0.23, *p* = 0.02) and age (*r* = 0.21, *p* = 0.03).

## Discussion

### A Nomenclature for Distinguishing Between Anesthetic Maintenance States

**Figure 9:**
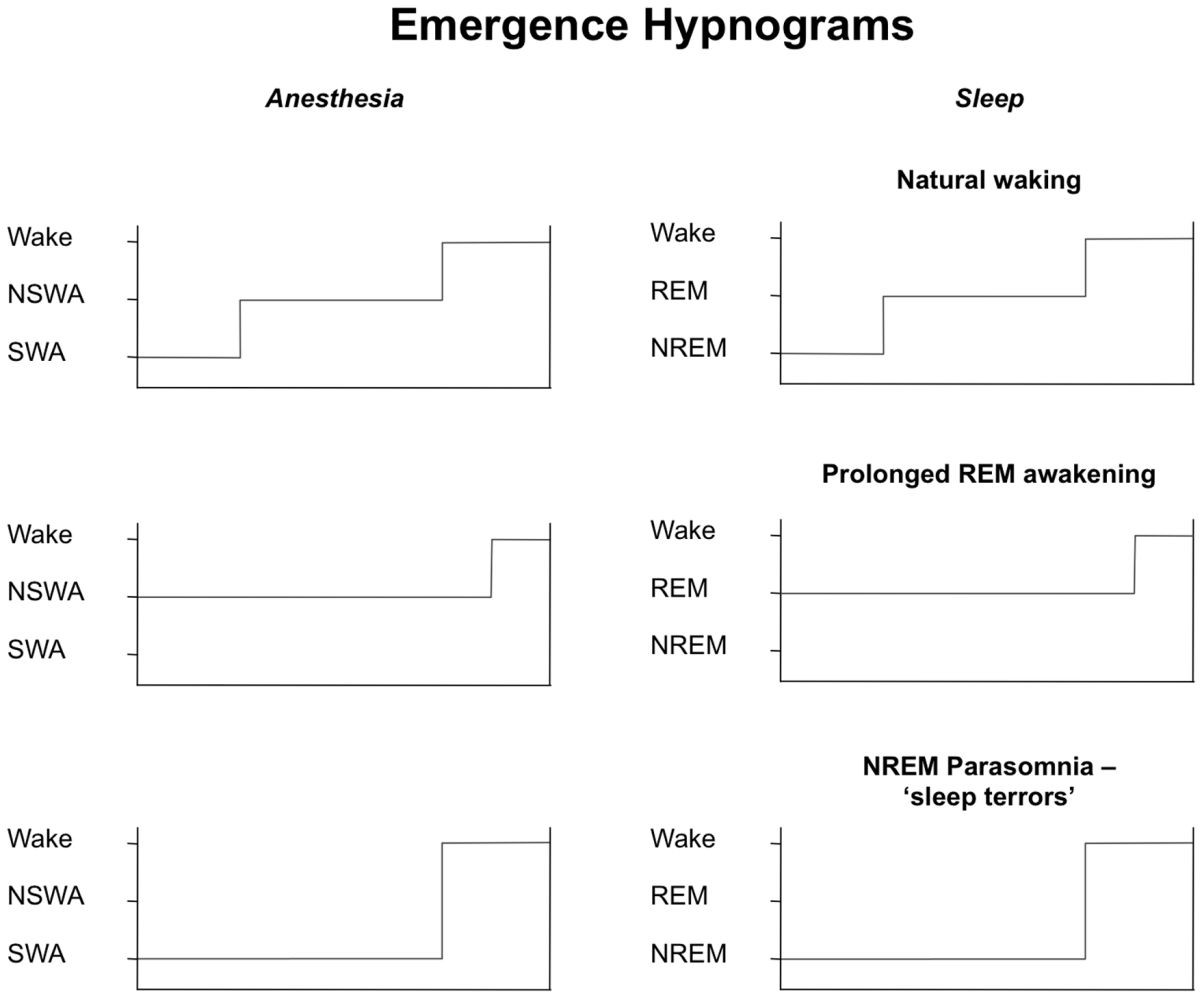
Hypnograms for emergence from general anesthesia and sleep. Temporal evolution of stages of arousal during emergence from general anesthesia (GA) are plotted in the left column, and from sleep in the right column. Each GA hypnogram on the left reflects an identified Emergence Trajectory as defined earlier in the text. These trajectories might be loosely correlated with arousal trajectories from various stages of sleep that are placed immediately to the right. SWA  =  Slow-Wave Anesthesia, NSWA  =  Non Slow-Wave Anesthesia; NREM  =  non-REM sleep, REM  =  REM sleep.

In this study, we present a conceptual framework to discriminate four patterns of anesthetic maintenance comprised of two states, ‘Slow-Wave Anesthesia’ (SWA) and ‘Non Slow-Wave Anesthesia’ (NSWA), and two derivative sub-classes of the slow-wave state, ‘delta-dominant’ (ddSWA) and ‘spindle-dominant’ (sdSWA). These classes were defined by the relative contribution of delta (0.5–4 Hz) and spindle-alpha (8–14 Hz) power in their spectral signatures (Figure 2B). These sub-14 Hz oscillations that dominate the EEG during both NREM (slow-wave) sleep and general anesthesia have been shown to correspond to diminished responsiveness to mild stimuli in NREM sleep [41], [61] and to more aversive stimuli during general anesthesia [9], [62], [63]. Because these oscillations can also be correlated with specific neural generators in a more generally hyperpolarized reticulothalamocortical circuit, they have been used for decades to classify stages of natural sleep. We were therefore able to capitalize on the methods by which sleep is staged and dynamically tracked over time (Figures 1, 9) to generate a new standardized taxonomy that could be used to describe the maintenace phase of general anesthesia. Although a pattern similar to the spindle-dominant slow-wave pattern (sdSWA) has been previously reported in studies in which volunteers are exposed to slowly changing levels of propofol anesthesia [8], [64], the greater diversity of patterns we observed may be due to the lack of spectral averaging in our subjects, the greater diversity of the typical surgical population (e.g. baseline neurological status and co-morbidities, pre-operative medication status, baseline pain sensitivity), and the highly variable surgical environment (e.g. intraoperative medication administration, surgical stimulation, nociception).

### A Description of Emergence Trajectories

We examined the transition from unconsciousness back to conscious awareness (i.e. responsiveness) after general anesthesia was terminated at the conclusion of surgery, from End Maintenance/Start Emergence to End Emergence. We found a general shift from the slow-wave pattern (SWA) to a more uniform distribution of power across frequency bands (NSWA). This trend was immediately visible and highly significant when the grand average of EEG spectra across the entire patient population was performed at the beginning and end of emergence (Figure 2E, p<0.05). However, just as with the maintenance patterns, individual patient emergence trajectories from unconsciousness to waking were more variable than averaged trajectories [8], [9] (Figure 2F, Figures 3–6). We speculate that inactivation of endogenous sleep networks, activation of redundant arousal networks, and the nociceptive residuum experienced by the patient, contribute to this variability. In order to minimize non-surgical noxious stimuli, an LMA was used as the airway device, and auditory and tactile stimulation during emergence was limited (see Methods); we therefore do not attribute this variability to tracheal or general environmental stimulation. More importantly, these diverse emergence patterns suggest that recovery from general anesthesia does not necessarily mirror the induction process, and may reflect a behavioral state barrier, which depends in part on the balance of activation in endogenous sleep and arousal pathways [31], [65]–[67].

Using a dynamical systems approach, we grouped 100 observed emergence trajectories in state-space into categories based on the apparent number of attractors during emergence, the time each subject occupied that stable attractor state, and the time required to transition between attractor states (Figures 3–6). As with any empirical study, the exact dynamical system classification of the attractor topology is provisional. Although the attractors resembled simple point attractors, more detailed studies of the EEG dynamics might reveal some high dimensional chaotic or limit cycle structure within these attractors. With those provisos, almost half (43%) of patients exhibited a canonical sequence: loss of delta, followed by loss of spindles, and then a period of NSWA (characterized by the absence of low frequency oscillatory peaks in the EEG and behavioral unresponsiveness), before waking. In contrast, approximately one-third (31%) of subjects skip the NSWA stage and transition directly from SWA to wakefulness (Trajectory 4), and 16% do not achieve SWA at any stage during end maintenance or emergence (Trajectory 3). We also observed a sharp increase in electromyographic power (EMG) that often precedes the return of consciousness, but is not a necessary pre-requisite.

### Connections between Emergence Trajectories and Post-operative Recovery

The time spent in NSWA prior to wake-up (defined on the dwell-time plots from Figures 3–6) was modestly predictive of subsequent pain (Figures 7–8). Patients who spent little of their emergence time in NSWA, were more likely to have high pain, and express a degree of agitation in recovery. (A similarly sudden transition from slow wave sleep directly to wakefulness is associated with some sleep pathologies such as night terrors [53], [68], see also FIgure 9). These patients often had more frontal EMG activation during emergence, which in itself was weakly associated with a higher pain score in the PACU. Frontalis muscle activation (EMG) in patients can in itself confound the results. The EMG signature is broad-band and can potentially contaminate, distort or obscure the underlying EEG signal. Conversely EMG activation is a valuable indication of motor system activation as part of the emergence sequence from anesthesia, and we found it to be largely independent of the return of consciousness, as defined by recovery of responsiveness to voice. This observation is in accord with other work suggesting that components of motor systems are relatively resistant to the effects of anesthesia, and that recovery of motor tone is not captured by changing information in the EEG which tends to reflect the hypnotic axis [69]. Different central nervous subsystems may not always reconnect in a well-defined sequence after general anesthesia is discontinued; in some instances, alternate paths or sequences for bringing various brain arousal nuclei or networks on-line preceding the return of consciousness may predispose patients to undesirable wake-ups, in the same way that parasomnias are exacerbated by disrupted sleep architecture [54]. Our measurements also do not assess recovery along the axis of memory, another subsystem affected by anesthetics and sleep.

### The evolution of trajectories from Start to End Emergence can be captured in an emergence hypnogram for general anesthesia

Figure 9 graphically depicts the evolution of each of the emergence trajectory patterns (Figures 3–6) and compares these patterns to arousal from specific stages of sleep. Carrying the analogy between waking from natural sleep and emergence from general anesthesia further, we propose using a hypnogram to capture the emergence trajectories from general anesthesia (left column) seen in Figures 3–6, where the length of the horizontal line at each stages reflects the relative dwell-time in the state-space plot (panel C of Figures 3–6). These can be compared to the sequences of emergence from natural sleep, seen in the right column, in the more classic version of the emergence hypnogram. A transition from SWA to NSWA before waking may correspond most closely to the waking from natural sleep. Waking from very prolonged NSWA may not have a correlate in normal sleep (prolonged REM awakening), but may instead reflect certain hypersomnia subtypes [70]. The emergence trajectory of an abrupt transition from SWA to wakefulness can be compared to arousal during NREM sleep, often associated with parasomnias or sleep disturbances resulting from fragmented sleep [53], [54].

### The Neurochemical Relationship Between Sleep Circuitry And General Anesthesia

**Figure 10:**
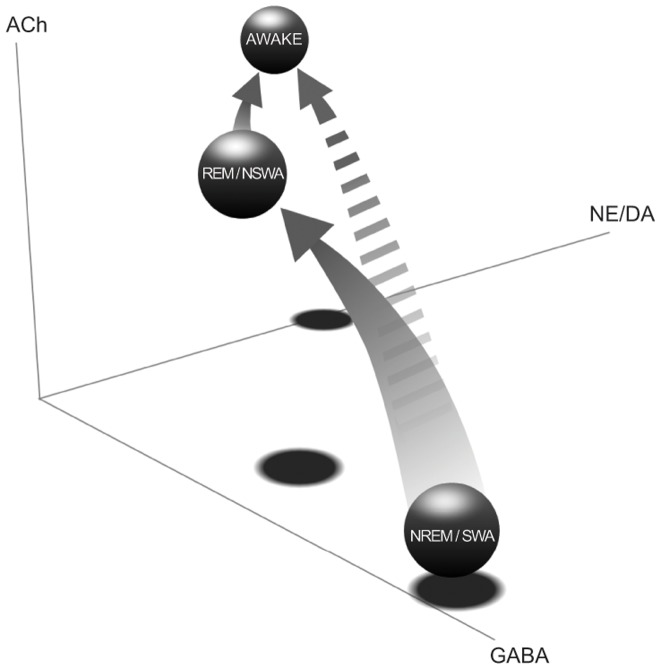
A putative (shared) 3-D neurotransmitter state space for emergence from sleep and general anesthesia. Based on spectral power and underlying neural generators, stages of anesthesia and stages of sleep may be analagous, especially on the path from thalamocortical hyperpolarization to waking. During natural NREM sleep, levels of GABA are high, while REM is characterized by high levels of both GABA and acetylcholine (ACh). As the brain passes from REM to waking, levels of GABA diminish, and monoamines such as norepinephrine and dopamine start to increase. The progression on the more typical anesthetic-emergence trajectory from SWA -> NSWA -> waking may reflect a similar shift in neurotransmitter balance as shown in this diagram (solid arrow). The “non-preferred” pathway of emergence that transitions directly from SWA -> waking (somewhat analagous to NREM -> waking) is represented by the broken arrow. NREM  =  non-REM sleep, REM  =  rapid eye movement sleep, SWA  =  Slow-Wave Anesthesia, NSWA  =  Non Slow-Wave Anesthesia, GABA  =  GABA-aminobutyric acid, ACh  =  acetylcholine, NE  =  norepinephrine, DA  =  dopamine.

The degree of thalamocortical hyperpolarization depends on the balance of activity in neurotransmitters systems [71], [72]. While there are many regional and nuclear brain differences, NREM sleep tends to be dominated by high GABAergic and low cholinergic and monoaminergic tone, while REM sleep is dominated by both GABAergic and cholinergic tone, and low levels of monoamines [73], [74]. We postulate that the presence of spindles and delta oscillations within the stage we define as SWA also reflects overwhelming GABAergic tone and low cholinergic tone, consistent with the mechanism of action of many of our volatile and intravenous anesthetics. The exact sequence of neuromodulator activation during emergence from anaesthesia is unknown, and is probably the main cause for the heterogeneity that we observed in the emergence trajectories. As anesthetic washes out of the brain, the residual GABAergic effects of the drug may be antagonized by depolarizing cholinergic (or aminergic) systems, pushing the brain into the NSWA state. The reason for transition from this point to waking is unclear, but probably involves a switch from high GABAergic tone to high monoaminergic and orexinergic tone, allowing the patient to fully emerge and reconnect with the external world. These pathways may be visualized in a 3-D neurotransmitter diagram for NREM/SWA, REM/NSWA, and waking states (Figure 10). The solid arrow signals a “preferred” wake-up sequence in which a patient moves from SWA to NSWA prior to waking, and experiences a higher degree of conscious awareness and a lower degree of pain and agitation in recovery (i.e. the classic cycle of NREM to REM to waking). The “non-preferred” sequence – in which the brain transitions directly from SWA to waking state – is reflected by the broken arrow, which if compared to sleep, would characterize a parasomnic or less desirable trajectory to re-establishing conscious awareness.

### Underlying Neurobiology of EEG Signatures of Emergence and their Relationship to Sleep

Both sleep and general anesthesia share many similarities, including specific nuclei, circuitry, and neurotransmitter systems. Physiological recordings [75]–[76] and modeling [64], [77] suggest that the oscillatory components of the alpha band (spindles between 8–14 Hz) in the frontal EEG during sleep and anesthesia are generated by hyperpolarizing neurons of the reticular nucleus of the thalamus, in the permissive environment of depressed ascending cholinergic and monoaminergic brainstem activity [75], which switches them to a burst firing mode [78]–[80]. Spindles predominate in NREM stage 2 sleep, and are more commonly observed during general anesthesia when analgesics are administered to diminish noxious stimulation [63]. NREM stage 3 sleep reflects an even greater degree of thalamocortical hyperpolarization as spindle activity diminishes and slower delta oscillations (0.5–1 Hz) begin to dominate [40]. Because our data demonstrate a positive correlation between anesthetic dose and relative delta power [81] we suggest that NREM stage 3 sleep may be similar to a ddSWA pattern under anesthesia, and NREM stage 2 sleep to an sdSWA pattern. This notion is further supported by the observation that at the midpoint of emergence (with decreasing anesthetic dose) there is an increase in the number of subjects categorized as sdSWA.

A progressive depolarization within the corticothalamic system accompanies the ordered transition to waking from natural sleep. This state is termed REM and is typically the stage of sleep from which most individuals awaken. Similarly, most subjects emerging from general anesthesia appear to move progressively from SWA to NSWA. Can NSWA be likened to REM sleep? Approximately 25% of patients report dream mentation if queried immediately upon waking, and it was more common for the group who reported dreaming to be in NSWA (i.e. have low power in the <14 Hz range) prior to waking [55]. Thus, SWA and NSWA may directly correspond to different levels of activation in the two distinct networks of conscious awareness (i.e. awareness of environment and awareness of self) described by others [82]. NSWA may reflect an unresponsive state in which some form of internally-directed consciousness has been established (e.g. fronto-parietal connectivity), but there is still insufficient brain function to connect externally. This is consistent with the concept of an unconnected (i.e. with the external world) or covert consciousness, which has been seen using carefully controlled propofol infusions in human volunteers with combined fMRI/EEG [9]. The thalamocortical network is rendered insensate to external inputs, yet activation to stimuli is still observed in cortical areas such as the precuneus. The role of the thalamus is less clear. It plays an important role in sleep, but its contribution to anesthesia has been questioned [83], and thalamic deactivation may not be the cause of unconsciousness but rather a consequence, via a functional thalamic de-afferentation from diminished cortical connectedness [84], [85].

One significant limitation to this study is that we only used a frontal EEG electrode strip for our recordings. Our data is therefore constrained to data gathered from accessible frontal leads (corresponding approximatley to FPz, FP1/2 from a traditional 10–20 montage) without simultaneous recording from parietal, temporal or occipital regions. We therefore could not consider other markers of loss and return of consciousness such as global coherence [8], [16] or the degree of connectedness and communication between different cortical regions [11], [13]–[15], [86]. However, EEG power is largely anteriorized upon loss of consciousness under general anesthesia [11], [16], [17], [87], making the frontal leads reasonable markers for spectral changes that can be observed at the single electrode level. Use of commercially available EEG electrodes that can be easily placed on a patient's forehead intraoperatively as part of the placement of routine monitors also increases the likelihood that our methods for tracking and staging depth of consciousness could be used more routinely by clinicians making real-time decisions in the operating room. Our data indicate that a long period of NSWA before waking is associated with less pain than a direct transition from SWA to waking. Without further prospective randomized clinical studies, it is unclear whether it would actually be clinically useful to specifically titrate anesthetic (or analgesic) drugs to target any particular intraoperative spectral pattern. Apart from the effect of age on spectral power, we found relatively weak correlations between clinical variables and spectral pattern. This would suggest that the spectral pattern for each patient is largely determined by other unknown (possibly inherited) factors. Nevertheless we would hope that this study provides a methodological framework in which these questions could be answered.

## Conclusions

The unconsciousness that accompanies general anesthesia during surgery is marked by several, distinct, oscillatory patterns in the EEG. Because these share features with those observed in natural sleep, it is possible that the underlying neural generators are conserved. Building upon a classification system used to stage sleep, we provide, for the first time, a standardized EEG-based nomenclature by which anesthetic maintenance can be staged and followed intraoperatively. We also applied a dynamical systems approach to characterize the return to consciousness after discontinuation of the anesthetic. We found that recovery of consciousness varies amongst individuals, and that deviations from a canonical sequence of SWA to NSWA occur. Further, the functional path by which the brain re-establishes conscious awareness is correlated with preferred wake-ups (e.g. diminished pain). We have created a descriptive means by which endogenous sleep rhythms and general anesthesia can be compared. Subsequent usage of this nomenclature should further our understanding of the overlapping neural mechanisms of sleep and anesthesia. As the homeostatic, learning and memory benefits of sleep are beginning to be characterized [88], future work may identify targets to improve post-operative cognitive health. The latter is of increasing concern as patients, researchers, and physicians become more apprehensive about the potential for anesthesia to cause or exacerbate adverse neurocognitive sequelae.

## Methods

### Ethics Statement

Studies were approved by the Northern Y Regional Ethics Committee of New Zealand (NTY/11/EXP/077), and written informed consent obtained from each patient. Free and public access to the de-identified data is available from the website: www.accesshq.org.

### Patient Selection

We studied 100 adult patients presenting for routine orthopedic surgery under general anesthesia at Waikato Hospital, New Zealand, from November 2011 to January 2013. Data collection was restricted to patients eligible for laryngeal mask airway (LMA) management, considered less stimulating than an endotracheal tube [89], in order to minimize confounding effects of airway irritation, a strong non-surgical arousal stimulus, on the EEG. We specifically included patients who would be expected to have a wide range of post-surgical pain levels (some augmented with regional nerve blocks). We excluded patients with pre-existing psychiatric illness, chronic substance abuse, chronic pain, or obesity (BMI>35). Patient demographics are represented in Table 2.

**Table 2:** Demographic Data.

**Age (yrs)**	48(19)
**Gender (F/M)**	48/52
**Duration of surgery (min)**	99(68)
**Duration of emergence (min)**	12.4 [8.4 to 18.9]
**Sevo/Des/Isoflurane**	81/8/11
**C_e_MAC_s_**	10.79 [0.66 to 0.98]
**C_e_MAC_e_**	0.06 [0.001 to 0.08]
**C_e_fentanyl_e_ (ng/ml)**	0.54 [0.31 to 0.78]
**PACU-Cons (0/1/2)**	17/42/41
**PACU-Pain (0/1/2)**	56/15/29

C_e_ denotes effect site concentration of the drug, and the subscripts ‘s’ and ‘e’ denote the start and end of emergence. MAC indicates units of age adjusted Minimum Alveolar Concentration, PACU-Cons and PACU-Pain are defined in the text and in Table 1.

### Surgical Procedure and Data Collection

In order to determine the spread of emergence patterns at the conclusion of routine clinical anesthesia, we did not restrict the course of administration of the general anesthetic with the exception of limiting the airway device to an LMA, allowing it proceed according to the clinician's judgment. Patients were induced with a bolus dose of intravenous fentanyl (50–200 µg) followed by propofol (80–200 mg). Patients were maintained on a volatile general anesthetic (VA) of the clinician's choice - sevoflurane, desflurane, or isoflurane - delivered in a mixture of air and oxygen. No neuromuscular blocking drugs were used. Intraoperative analgesia was provided by intravenous morphine (0–25 mg); analgesic adjuncts of paracetamol (1 gm, n = 34), parecoxib (40 mg, n = 39), tramadol (100–200 mg, n = 22) or clonidine (60–120 µg, n = 18) were given as needed. 21 patients received a peripheral nerve block for post-operative analgesia; 14 of these were femoral blocks, done for reduction of postoperative muscle spasm for lower limb joint replacements. Because of the mutineuronal innervation of the knee and hip, most of these nerve blocks achieved incomplete analgesia. Of the others (mainly ankle blocks and brachial plexus blocks), 5 patients had pain scores of 0 on awakening – indicative of a completely successful block.

We used standard American Society of Anesthesiology [90] monitors (pulse oximeter, non-invasive blood pressure cuff, electrocardiogram, capnograph) to measure intraoperative oxygen saturation, hemodynamics, and end-tidal gas concentrations (O_2_, CO_2_, VA). In addition to these routine parameters, the times of surgery, tourniquet inflation and release, and drug administration were recorded.

A 2-lead EEG prefrontal electrode strip was placed on the patient's forehead, as per the manufacturer's recommendations (BIS, Covidien Vista). Frontal EEG data (sampling rate 128 Hz) was collected for the duration of the anesthetic, prior to induction through the end of emergence. The frontal EEG waveforms on the BIS sensor strip correlate approximately to the FPz and FP1/2 leads of standard 20 electrode EEG montage. Both electrodes are referenced by an additional electrode over the eyebrow. A separate lead over the temporalis muscle is used as an EMG electrode. Suitable electrode impedance (<5 kOhm) was confirmed using the manufacturer's automatic checking routine. To provide a mechanism to correlate external events with the patient's EEG, the times of surgical incision, cessation of the volatile anesthetic agent, and the exact moment of awakening were marked on the EEG record by tapping the electrodes 4 times in ∼1.5 seconds to create a clear stimulus artifact. The patients were allowed to waken without excessive stimulation beyond the usual noise and activity of the operating room and post anesthesia care unit (PACU) environment (e.g. removal and replacement of monitors, background talking). Sustained awakening was confirmed by a positive response to verbal command (“Mrs. X, your operation is over, open your eyes.”). ‘Emergence’ from general anesthesia was specifically defined as the time between the cessation of administration of the maintenance hypnotic drug (VA) and the point at which the patient became responsive to verbal command. Raw EEG and EMG data for each patient were downloaded post-operatively for further analysis.

### Drug Effects

The decreasing effect-site concentrations (*C_e_*) of the different volatile anesthetic drugs were estimated as a fraction of age adjusted MAC [91] at Start and End Emergence, and the estimated effect-site concentrations of the intraoperative opioids (fentanyl and morphine) were calculated using standard compartmental pharmacokinetic modeling [92], [93]. In order to compare opioid effects across patients, and to take into account the fact that many patients had received both morphine and fentanyl intraoperatively, we expressed the morphine concentrations as fentanyl concentration equivalents (*C_e_ fentanyl*), assuming a conversion factor of 50. This data is also summarized in Table 2.

### Evaluation of post-emergence level of consciousness and pain

It is common for patients to experience fluctuations in both level of consciousness and pain in the early postoperative period. These changes are hard to capture and analyze [94]. Accordingly we used simple, heuristically derived, descriptions of level of consciousness (PACU-Cons) and pain (PACU-Pain) as described in Table 1. We believe that these classifications have the advantage of capturing the clinically relevant, qualitative aspects of the recovery period. The PACU-Cons score is based on the change in the Ramsay Score over the first 15 minutes in the PACU [95]. The severity of the pain was quantified using a composite of the standard integer numerical verbal rating scale (NRS) – where 0 is no pain and 10 the worst imaginable pain. Because of the questionable validity of the fine gradations in these semi-quantitative scores, the PACU-Pain score bins the numerical rating scale into ‘minimal’, ‘moderate’ and ‘severe’ pain groups [96], which appear to have reasonable clinical validity. However, we also scored the degree of objective distress that the patient manifested, and used this added information to modify these scores as described in Table 1. The first pain score was obtained when the patient had achieved a level of consciousness at which they could maintain a brief conversation. These pain queries were then repeated every 15 minutes. If they were in pain, patients were treated with increments of 1–2 mg IV morphine, 25–50 µg of fentanyl, or 50–100 mg IV tramadol in the PACU, and then discharged to the ward according to institutional criteria based on the Aldrete score [97].

### Spectral Processing, Statistical and Analytical Methods

All data was analyzed using custom scripts written in MATLAB (Mathworks, Natick, Massachusetts). The amplitude and frequency structure of the EEG signal was initially described by the mean power in the traditional frequency bands: delta (0.5–4 Hz), theta (5–7 Hz), alpha (8–14 Hz), and beta (15–25 Hz). The power in each band was then logarithmically transformed so that all spectral power was expressed in units of dB (10× logarithm base 10, referenced to 1 µV^2^/Hz). These band-powers were then smoothed using a 30 second median smoothing filter. In addition we recorded the EMG output (dB) from the Vista monitor. We then extracted EEG features (the delta and alpha bands) that reflected the largest changes in spectral power seen in the prefrontal channels during emergence. Spindle power was specifically derived by measuring the height of the alpha peak above the underlying broadband activity to target the narrowband oscillatory component of this peak [55].

To follow these changes dynamically, we displayed the EEG using typical spectrograms and time series plots of salient features (spindle, delta and EMG power). Spectrograms of the EEG data were made using the ‘mtspecgramc.m’ function and PSDs using the ‘mtspectrumsegc.m’ function of the Chronux toolbox (http://chronux.org) [98] on 8 second segments, with 6 second (75%) overlap and tapers of 13 and 8. This segment length was chosen to balance the time required to reliably estimate power in the delta band, while still being short enough to allow for changes in the detection of waveforms within a clinically relevant time frame. 95% confidence intervals for the median PSD for each condition (Start and End Emergence) were calculated using a jackknife estimation method. To create 3-D individualized dwell-time plots for each patient's emergence trajectory (Figures 3–6, panel C), spindle and delta power were plotted against one another in x-y plane, while depth on the z-axis reflected the total amount of time a patient remained in each x-y pixel of the spindle-delta state space as a fraction of total emergence time. This allowed us to capture the changing relationship between oscillations over time, and permitted comparisons across different patients with differing lengths of emergence.

Correlations between pairs of variables were quantified using Pearson's correlation co-efficient (r) if they followed a normal distribution (Kolmogorov-Smirnoff test), otherwise Spearman's rank correlation was used. The multivariate analysis of the interrelationships between all the variables in an observational study is subject to unknown confounding factors. Following conservative statistical practice, we limited the total number of explanatory variables for each model to 5, in order to have 20 data points to estimate the parameter for each variable with reasonable precision. We used multiple linear regression models, with stepwise forward selection, to discover which group of variables were most significantly associated with the continuous outcome variables: namely delta and alpha power at Start Emergence, and emergence time (which was logarithmically transformed to achieve a normal probability distribution). We used similar techniques, but using logistic regression, for binary outcome variables (pain group).

A patient's post-operative nociceptive status was loosely correlated with the emergence trajectory. In order to display those average trajectories, we combined the phase-space data (spindle vs. delta power) for all patients in the low (PACU-Pain  = 0) and high (PACU- Pain  = 2) groups (Figure 8). More stereotypical patterns were reflected in data points remaining tightly constrained in the 2-D phase space. More variable EEG emergence trajectories were reflected in points spread out over a large area of the 2-D phase space. Cumulative time spent in each area of the phase space was reflected in a color heat map from blue (little time) to red (large amount of time).

## Supporting Information

Figure S1 **This 3-D histogram of spindle and delta power at Start Emergence, calculated on a single 8 second epoch for each patient (z-axis), reveals this data follows a unimodal distribution.** Following statistical convention, patients were separated into two main spectral classes by thresholding spindle and delta power at 7 dB. This theshold reflects the boundary below which only 5% of the Start Emergence data lay, and the region within which most of the End Emergence data lay. Patients who began emergence (i.e. at the termination of general anesthetic delivery) above the 7 dB threshold (above the gray box) were termed to be in Slow-Wave Anesthesia (SWA). Those within the gray box, or below 7 dB, were termed to be in Non Slow-Wave Anesthesia (NSWA).
(TIF)
