# Supplementary figures and images for: Electroencephalographic Variation during End Maintenance and Emergence from Surgical Anesthesia
Source: PLoS One. 2014 Sep 29;9(9):e106291. doi: 10.1371/journal.pone.0106291 (PMC4180055; doi:10.1371/journal.pone.0106291)

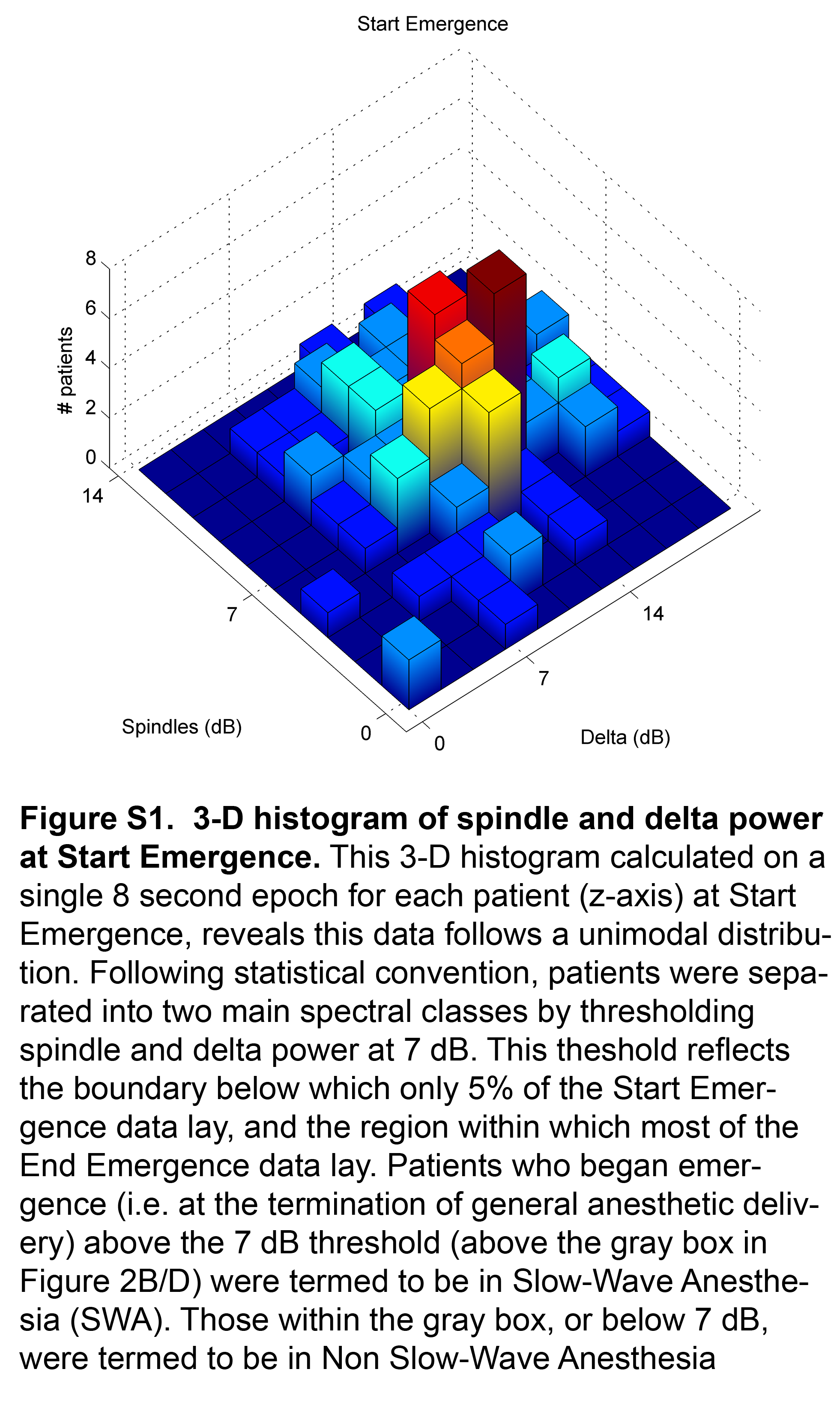

Supplement: Figure S1 — This 3-D histogram of spindle and delta power at Start Emergence, calculated on a single 8 second epoch for each patient (z-axis), reveals this data follows a unimodal distribution. Following statistical convention, patients were separated into two main spectral classes by thresholding spindle and delta power at 7 dB. This theshold reflects the boundary below which only 5% of the Start Emergence data lay, and the region within which most of the End Emergence data lay. Patients who began emergence (i.e. at the termination of general anesthetic delivery) above the 7 dB threshold (above the gray box) were termed to be in Slow-Wave Anesthesia (SWA). Those within the gray box, or below 7 dB, were termed to be in Non Slow-Wave Anesthesia (NSWA). (TIF) [file pone.0106291.s001.tif]
